# Current Unemployment, Unemployment History, and Mental Health: A Fixed-Effects Model Approach

**DOI:** 10.1093/aje/kwac077

**Published:** 2022-04-20

**Authors:** Liina Junna, Heta Moustgaard, Pekka Martikainen

**Keywords:** hospitalization, longitudinal study, mental health, unemployment

## Abstract

Poor mental health among the unemployed—the long-term unemployed in particular—is established, but these associations may be driven by confounding from unobserved, time-invariant characteristics such as past experiences and personality. Using longitudinal register data on 2,720,431 residents aged 30–60 years, we assessed how current unemployment and unemployment history predict visits to specialized care due to psychiatric conditions and self-harm in Finland in 2008–2018. We used linear ordinary-least-squares and fixed-effects models. Prior to adjusting for time-invariant characteristics, current unemployment was associated with poor mental health, and the risk increased with longer unemployment histories. Accounting for all time-invariant characteristics with the fixed-effects models, these associations attenuated by approximately 70%, yet current unemployment was still associated with a 0.51 (95% confidence interval: 0.48, 0.53) percentage-point increase in the probability of poor mental health among men and women. Longer unemployment histories increased the probability among men in their 30s but not among older men or among women. The results indicate that selection by stable characteristics may explain a major part of the worse mental health among the unemployed and especially the long-term unemployed. However, even when controlling for this selection, current unemployment remains associated with mental health.

## Abbreviation

CI: confidence interval
FE: fixed effects
LPM: linear probability model
OLS: ordinary least squares

Unemployment is associated with poor mental health. The unemployed—the long-term unemployed in particular (1–3)—exhibit higher levels of distress, psychiatric symptoms, and self-harm than the employed (1–11). However, it remains unclear to what extent the relationship between unemployment and mental health is causal. Unemployment could harm mental health through mechanisms such as increased stress following the loss of income, social contacts, and meaningful activities associated with employment (1, 6). These hardships also accumulate over time, which may explain why longer periods of unemployment have been found to be more detrimental to health than shorter spells (1–3). On the other hand, poor mental health may increase both the risk of becoming unemployed and longer unemployment spells through health-related selection (12–17). These explanations are not mutually exclusive. The poorer mental health among the unemployed likely reflects a complex interplay between health and unemployment (18).

A large share of the observational studies that suggest unemployment is detrimental to mental health have dealt with selection and confounding by adjusting for preexisting mental health problems and various other personal and contextual characteristics associated with unemployment and mental health (2, 5, 6). Some of these confounders—such as age, marital dissolution, and the labor market context (best measured as time-varying) and maternal language and baseline education (time-invariant)—are relatively easy to address (2, 3, 5, 6). However, recent studies have suggested that a notable share of the association could be due to confounding by stable individual characteristics such as health histories (1), genetic background and childhood conditions (19–23), early career experiences (24, 25), and personality (26–30). These complex characteristics are demanding to measure accurately and therefore to fully control for.

Some studies have used the fixed-effects (FE) approach to tackle time-invariant confounders (31–38). In FE models, the individual is compared with him- or herself over multiple measurement points with differing exposures, which removes all confounding by time-invariant characteristics. The results from these studies are inconsistent: Unemployment has been found to be detrimental among the general population (36, 37), only among subpopulations unemployed due to reasons other than workplace closure (32), or those below the age of 30 years (31, 33, 38). Variability in these results is likely to relate to measurement or methodological choices, adjusted time-varying characteristics, or differences in study context and policy environments.

Moreover, FE studies have primarily addressed current unemployment while ignoring past experiences (31, 32). This is a notable gap in the literature. Observational evidence suggests that longer periods of unemployment are more strongly related to mental health (1–3). However, complex time-invariant characteristics such as personality are particularly important for selection into long-term unemployment and may therefore explain some of the prior findings (30, 31, 36). Furthermore, little is known about the contribution of time-invariant confounding across important demographic dimensions such as sex and age. In general, the effects of unemployment may be stronger among men, for whom employment is a more central social role (2, 36). It has also been suggested that unemployment could be more harmful to the middle-aged, with more financial responsibilities and stronger career commitments (5). Only a small number of FE studies have addressed unemployment histories or whether time-invariant confounders contribute similarly among men and women and different age groups (31, 36). The prior evidence is based on different outcomes and populations, and the results are conflicting. Mental health scores deteriorated with cumulative exposure to unemployment among Germans aged 30 or younger but not those older than 30 (men and women pooled together) (31). In turn, among Norwegians aged 18–67 years, impeding unemployment was associated with increased odds of initiating psychotropic medications, and the risk then remained elevated during the first months of unemployment, increasing slightly at 4 months (36). The estimates were slightly higher among men and did not differ between age groups. Whether time-invariant confounding plays a larger role among those above the age of 30 is therefore still unclear, as are the reasons behind this possible age heterogeneity.

We contribute to the literature by considering both current unemployment status and unemployment history as predictors of mental health. We quantified the total contribution of time-invariant confounding by using the within-individual FE design (34). We used data on the total Finnish working-age population in 2008–2018. High-quality general population data with long follow-up periods and objective mental health outcomes have previously been underutilized for this purpose (36). Register data are a notable strength when studying unemployment and mental health, as they do not suffer from problems related to loss to follow-up among individuals with poor mental health, or to misreporting sensitive measures. We additionally explored heterogeneity by age and sex.

## METHODS

### Data

The quarterly individual-level data on all Finnish residents aged 30–60 years and in the labor market (employed or unemployed) at some point in the years from 2008–2018 (*n* = 2,720,431) came from the population register of Statics Finland. We excluded all individuals aged below 30 years as the share of students in these ages is large (39), and we could not reliably identify students. We identified all unemployment spells using onset dates from the Labour Market Data File of Statistics Finland. The included health conditions were derived from the Care Register for Health Care of the National Institute for Health and Welfare and the Death Records of Statistics Finland. Using the personal identification code that all Finnish residents receive, we then linked these data, along with sociodemographic information from Statistics Finland.

The focus of this study was comparing the unemployed with the employed, and we therefore excluded quarter-year observations outside the labor market (*n* = 6,560,568). Of all the excluded observations, 50% were in retirement, 15% were in education, and 35% were in other activities, such as taking care of the home. Consequently, individuals who were never in the labor market during the study period were excluded (*n* = 202,477). We also excluded observations with missing data on any of the measures, which led to excluding 12,164 (0.01%) of all observations. Individuals exited the sample by aging out, by migrating, by leaving the labor force permanently, or at death.

### Outcome

The outcome (*Yt*) was a binary measure of whether the individual experienced a mental health outcome (yes/no) over the quarter-year observation period (*t*). We considered visits to specialized care (booked and emergency inpatient and outpatient care visits) due to psychiatric conditions (*International Classification of Diseases*, *Tenth Revision* (ICD-10), main diagnosis, codes F01–F69) and self-harm (ICD-10, X60–X84) as proxies for poor mental health. As it is possible for a person to die due to mental health–related causes before reaching a hospital (e.g., suicides) we also included deaths related to the same causes (based on underlying cause of death).

### Exposures

Current unemployment was measured at *t* – 1 and was based on being registered as an unemployed jobseeker for a minimum of 2 days at any point during the quarter-year observation period (yes/no). Spells shorter than 2 days were not considered as unemployment because these are likely to represent workplace transitions. Unemployment history was measured as the cumulative number of observations with unemployment in the 3 years preceding *t* − 1. This was included in the models as linear and squared continuous measures, to allow for nonlinear associations (2, 31).

### Covariates

To adjust for time and age trends in visits to specialized care and unemployment, we used both birth year and calendar year as continuous measures and quarter as a binary measure; year and quarter were included as time-varying. A known protective factor is being in a relationship, as a partner may provide social and financial support as well as social control (1, 5). Partnership status was registered annually on December 31. We included it as a time-varying categorical measure (married or cohabiting, single, divorced, or widowed) for the preceding year. We further controlled for having coresident children (yes/no), also measured annually and included as time-varying.

### Statistical analysis

We estimated linear probability models (LPMs, ordinary-least-squares models for binary outcomes) for the association between quarterly current unemployment, unemployment history, and visits to specialized care. We modeled the association both with and without controlling for the unobserved, time-invariant characteristics using the FE estimator (34). As FE models rely solely on within-individual variance, this removes all between-individual contribution from any unchanging characteristics. The individual is used as his or her own control to compare deviations from the mean of unemployment status with deviations from the mean of the health outcome. The LPM-FE results can then be interpreted as the difference in the probability of the outcome among study subjects during periods of unemployment compared with periods in employment.

We fitted all models for men and women separately. The LPM model 1 was modeled separately for current unemployment and unemployment history, and adjusted for the calendar year, quarter, and year of birth to account for the initial age. In model 2, we simultaneously included current unemployment and unemployment history, adjusting for the same covariates as in model 1. The LPM-FE model 3 controlled for all time-invariant characteristics by design, and we thus excluded observed birth year from the model and again included current unemployment and unemployment history together. In the final model 4, we further adjusted for time-varying partnership status and having children. Model 4 can then be presented as

$$ {\displaystyle \begin{array}{l}{Y}_{it}={\beta}_1 CURRENT\_ UNEMPLOYMEN{T}_{it} \\ \kern2.45em +{\beta}_2 UNEMPLOYMENT\_ HISTOR{Y}_{it}\\ {}\kern2.25em +{\beta}_3 UNEMPLOYMENT\_ HISTOR{Y}^{2_{it}}\\ \kern2.45em+{\beta}_4 YEA{R}_{it}+{\beta}_5 QUARTE{R}_{it}\\ {}\kern2.25em +{\beta}_6 PARTNERSHI{P}_{it}+{\beta}_7 CHILDRE{N}_{it}+{a}_i+{\varepsilon}_{it}\end{array}} $$

where α is the fixed but unknown individual-level intercept, and ε denotes the error terms (35).

To estimate the probability of poor mental health for different levels of unemployment history by sex and age group, we further estimated an interaction between current unemployment and unemployment history for 10-year age groups, for whom we then calculated predicted probabilities. We fitted 2 interaction models: an ordinary-least-squares (OLS) model adjusting for year, quarter, and birth year (model 2 plus the interaction between current unemployment and unemployment history) and a FE model adjusting for year, quarter, partnership status, and coresident children (model 4 plus the interaction between current unemployment and unemployment history). The reference category was employed with no history of unemployment.

We estimated heteroscedasticity-consistent standard errors for all models (40). None of the models produced predicted probabilities outside the unit interval (0,1). Stata, version 16.1 (StataCorp LLC, College Station, Texas), was used for all analyses.

### Sensitivity analyses

To ascertain that our results were robust, we tested other cutoffs for current unemployment and unemployment history (Web Table 1, available at https://doi.org/10.1093/aje/kwac077) and fitted the models using logistic FE (Web Table 2).

## RESULTS

Table 1 describes the characteristics of the analytical sample. The maximum follow-up was 10 years (mean = 7.5). Men were unemployed 13.0% of the quarter-year observations and women 11.9%, while less than 1% of men and 1.3% of women had a mental health-related visit to specialized care (Table 1).

**Table 1:** Sample Characteristics, Men and Women in the Labor Force, Aged 30–60 Years, Finland, 2008–2018

**Characteristic**	**Men**		**Women**	
	**No.**	**% of Observations**	**No.**	**% of Observations**
No. of individuals	1,384,596		1,335,835	
Quarterly observations	41,908,188		40,428,174	
Age, years^a^	44.9 (8.9)		45.4 (8.9)	
Married or cohabiting		54.0		56.6
Has coresident children		51.4		56.1
Unemployed		13.0		11.9
Unemployment history, quarters^a^	1.3 (3.0)		1.2 (2.8)	
Visits to specialized care due to psychiatric conditions and self-harm	380,229	0.9	542,445	1.3

^a^ Values are expressed as mean (standard deviation).

Considered separately, current unemployment and a longer unemployment history were detrimental to mental health among both men and women (Table 2, Table 3; model 1): The probability of a visit to specialized care due to psychiatric conditions and self-harm was 2.21 (95% confidence interval (CI): 2.18, 2.25) percentage points higher among men during current unemployment and 1.98 (95% CI: 1.94, 2.02) higher among women. Each additional quarter of unemployment history was also associated with an approximately 0.3 percentage-point increase in the probability of a visit among both men and women. Considering current unemployment and unemployment history together attenuated the individual impact of both unemployment measures (Table 2, Table 3; model 2).

**Table 2:** Change in the Probability of Visits to Specialized Care Due to Psychiatric Conditions and Self-Harm Among Men Aged 30–60 Years^a^, Finland, 2008–2018

	**Model 1** ^**b**^		**Model 2** ^**c**^		**Model 3** ^**d**^		**Model 4** ^**e**^	
**Exposure**	**Coefficient**	**95% CI**	**Coefficient**	**95% CI**	**Coefficient**	**95% CI**	**Coefficient**	**95% CI**
Employed	0.00		0.00		0.00		0.00	
Unemployed	2.21	2.18, 2.25	1.51	1.48, 1.54	0.51	0.48, 0.53	0.50	0.48, 0.53
Unemployment history, quarters	0.29	0.28, 0.30	0.13	0.11, 0.14	−0.01	−0.01, 0.00	−0.01	−0.02, 0.00
Unemployment history^2^	−0.01	−0.01, −0.00	−0.00	−0.00, −0.00	0.00	0.00, 0.00	0.00	0.00, 0.00
ρ					46.54		46.49	

Abbreviation: CI, confidence interval.

^a^ Number of observations = 43,546,725; results presented in percentage points. Model estimates multiplied by 100.

^b^ Ordinary least squares, current unemployment, and unemployment history modeled separately. Adjusted for year, quarter, and birth year.

^c^ Ordinary least squares, current unemployment, and unemployment history modeled together. Adjusted for year, quarter, and birth year.

^d^ Fixed effects, year, and quarter.

^e^ Fixed effects, model 3 with the addition of partnership status and coresident children.

**Table 3:** Change in the Probability of Visits to Specialized Care Due to Psychiatric Conditions and Self-Harm Among Women Aged 30–60 Years^a^, Finland, 2008–2018

	**Model 1** ^**b**^		**Model 2** ^**c**^		**Model 3** ^**d**^		**Model 4** ^**e**^	
**Exposure**	**Coefficient**	**95% CI**	**Coefficient**	**95% CI**	**Coefficient**	**95% CI**	**Coefficient**	**95% CI**
Employed	0.00		0.00		0.00		0.00	
Unemployed	1.98	1.94, 2.02	1.47	1.44, 1.51	0.51	0.48, 0.53	0.51	0.48, 0.53
Unemployment history, quarters	0.31	0.30, 0.32	0.16	0.14, 0.17	−0.04	−0.05, −0.03	−0.04	−0.05, −0.03
Unemployment history^2^	−0.01	−0.01, −0.01	−0.01	−0.01, −0.01	0.00	0.00, 0.00	0.00	0.00, 0.00
ρ					47.60		47.58	

Abbreviation: CI, confidence interval.

^a^ Number of observations = 42,393,875; results presented in percentage points. Model estimates multiplied by 100.

^b^ Ordinary least squares, current unemployment, and unemployment history modeled separately. Adjusted for year, quarter, and birth year.

^c^ Ordinary least squares, current unemployment, and unemployment history modeled together. Adjusted for year, quarter, and birth year.

^d^ Fixed effects, year, and quarter.

^e^ Fixed effects, model 3 with the addition of partnership status and coresident children.

Controlling for all time-invariant factors (model 3), the association between current unemployment and visits to specialized care attenuated by approximately 70%. However, current unemployment still increased the probability of a visit by 0.51 (95% CI: 0.48, 0.53) percentage points among men and 0.51 (95% CI: 0.48, 0.53) among women. Including time-varying partnership status and having children (model 4) had little effect on the estimates.

**Figure 1:**
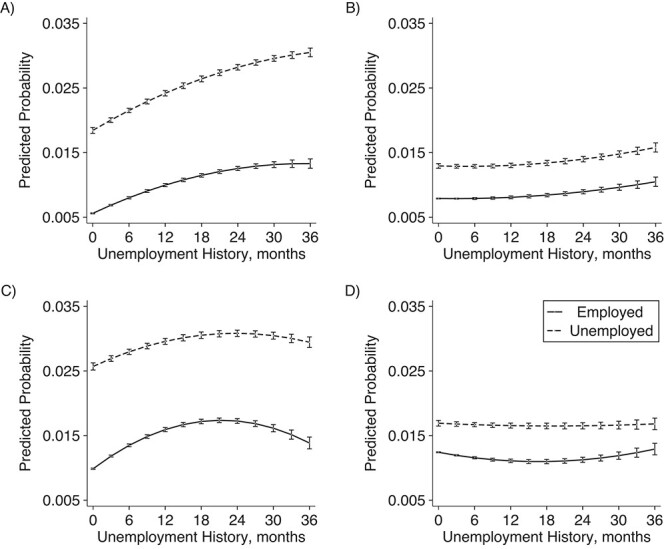
Predicted probability of visits to specialized care due to psychiatric conditions and self-harm among men and women aged 30–60 years, Finland, 2008–2018. Results are derived from an ordinary-least-squares (OLS) model adjusted for year, quarter, and birth year and a fixed-effects (FE) model additionally adjusted for year, quarter, partnership status, and coresident children: A) men, OLS model; B) men, FE model; C) women, OLS model; D) women, FE model.

We observed an interaction effect between current unemployment and unemployment history (Figure 1; Web Table 3). In the OLS models (Figure 1A and 1C) the combination of current unemployment preceded by a longer unemployment history was particularly harmful among men and, up to approximately 24 months, also women. However, unmeasured confounding due to time-invariant characteristics appears to explain a substantial share of the impact of both current unemployment and unemployment history: In the full FE models, the association between current unemployment preceded by a long unemployment history and visits to specialized care strongly attenuated among men and disappeared among women (Figure 1B and 1D).

**Figure 2:**
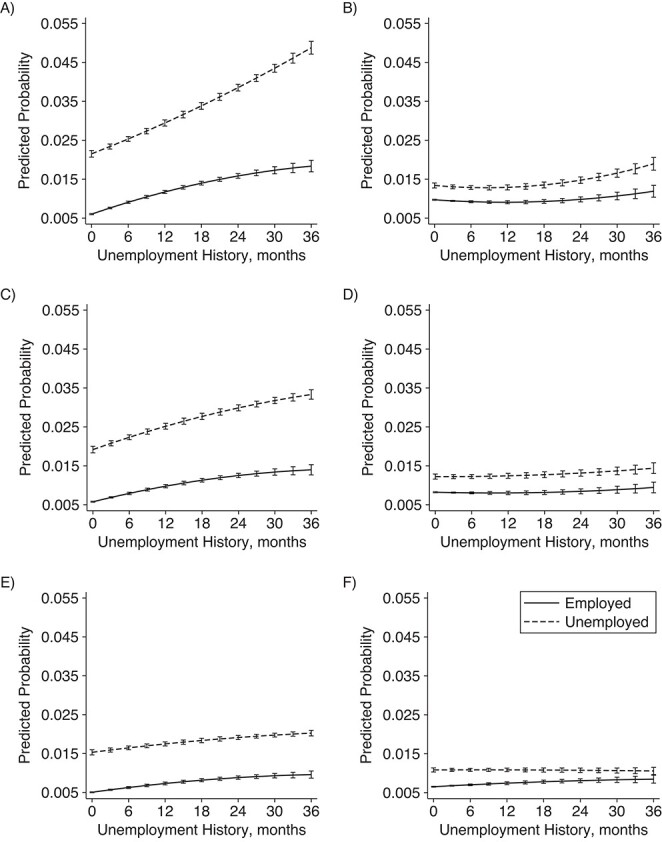
Predicted probability of visits to specialized care due to psychiatric conditions and self-harm among men according to 10-year age group, Finland, 2008–2018. Results are derived from an ordinary-least-squares (OLS) model adjusted for year, quarter, and birth year, and a fixed-effects (FE) model additionally adjusted for year, quarter, partnership status, and coresident children: A) OLS model, ages 30–39 years; B) FE model, ages 30–39 years; C) OLS model, ages 40–49 years; D) FE model, ages 40–49 years; E) OLS model, ages 50–60 years; F) FE model, ages 50–60 years.

**Figure 3:**
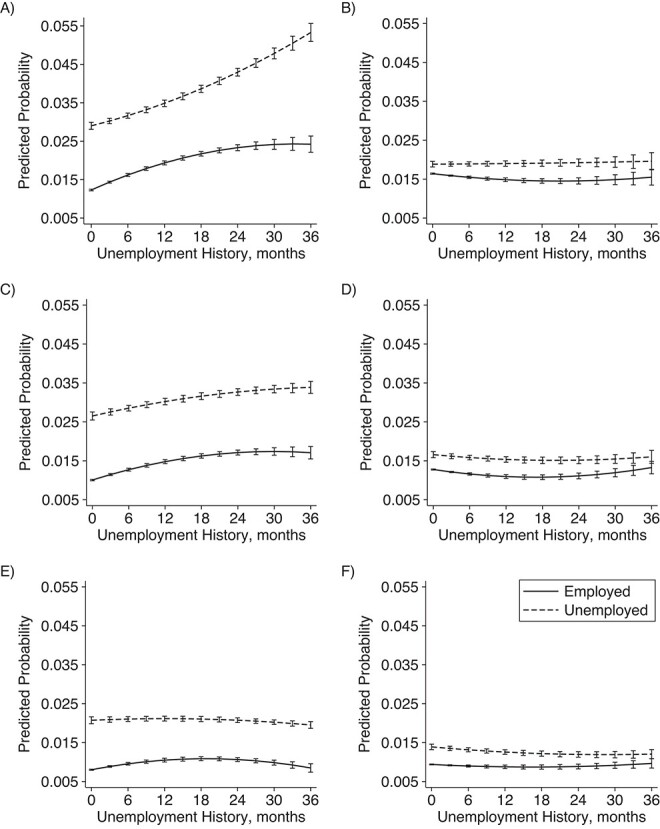
Predicted probability of visits to specialized care due to psychiatric conditions and self-harm among women according to 10-year age group, Finland, 2008–2018. Results are derived from an ordinary-least-squares (OLS) model adjusted for year, quarter, and birth year, and a fixed-effects (FE) model additionally adjusted for year, quarter, partnership status, and coresident children: A) OLS model, ages 30–39 years; B) FE model, ages 30–39 years; C) OLS model, ages 40–49 years; D) FE model, ages 40–49 years; E) OLS model, ages 50–60 years; F) FE model, ages 50–60 years.

The overall picture varied by age. In the OLS models, current unemployment preceded by a long unemployment history was particularly detrimental among men of all ages—but more so among the younger age groups—and women in their 30s and 40s (Figures 2–3; Web Tables 4–5). In the full FE models, this was observed only among men in their 30s.

In sensitivity analyses, our results were robust to different cutoff points of current unemployment and unemployment history (Web Table 1), as well as for model specification (Web Table 2).

## DISCUSSION

An extensive literature has demonstrated the association between unemployment and poor mental health (1–11). Whether this association is driven by confounding from time-invariant characteristics is important for understanding the determinants of mental health as well as the association between unemployment and health. We analyzed unemployment and visits to specialized care due to psychiatric conditions and self-harm using longitudinal register data of the total population of economically active Finns. In addition to addressing current unemployment, we assessed 3-year unemployment history to better understand the role of cumulative unemployment, and we further considered age- and sex-related heterogeneity. We used a fixed-effects design to control for all time-invariant characteristics such as personality, past experiences, and initial health status, that may confound the association between unemployment and health. To our knowledge, this is the first FE study on unemployment histories and mental health–related visits to specialized care.

We show that a large part—approximately 70%—of the association between current unemployment and mental health was due to confounding from time-invariant characteristics. Yet current unemployment among men and women and unemployment history among men were associated with an increase in probability of poor mental health even after controlling for this unmeasured confounding. The associations were modest: In the full models, current unemployment was associated with a 0.50 (95% CI: 0.48, 0.53) percentage-point increase in poor mental health among men and 0.51 (95% CI: 0.48, 0.53) percentage-point increase among women. However, the outcome is rare, and while the absolute difference was small, the relative difference was not. Even in the full models, the increase in the probability was 1.6-fold among unemployed men with no unemployment history and 1.4-fold among corresponding women when compared with the employed. In other words, insofar as our estimates can be considered causal, if unemployment increased by 100,000 men, we would expect 500 more mental health–related visits to specialized care, and if long-term unemployment increased by 100,000 men, we would expect 790 more visits (Web Table 4). These visits represent both adverse individual-level consequences and care costs to society. Moreover, visits due to psychiatric conditions and self-harm are likely to represent the tip of the iceberg. Less severe mental health measures, such as well-being scales, may react more strongly to unemployment than our fairly severe outcome (1).

### Current unemployment, unemployment history, and the role of causation and selection mechanisms

Based on prior observational evidence, unemployment lasting for 6 months or more could be more detrimental to mental health than are shorter spells (2, 5). This is plausible, as there are various material and psychosocial mechanisms through which longer exposure could be more damaging. Employment is an important source of income, and higher income in turn allows for personal choices that promote mental health, such as the use of health and leisure services. Financial insecurity is also stressful (41), and financial resources—both objective and perceived—are important predictors of mental health (42). The Finnish universal unemployment insurance system may somewhat soften the financial consequences of unemployment (43), but job loss is still typically followed by an immediate income drop, and subsequently another drop among the long-term unemployed once they exhaust their earnings-related benefits. During our study period, the more-generous earnings-related benefits provided by unemployment funds were available to those eligible (e.g., employed for a minimum of 26 weeks over the preceding 28) for a duration of approximately 300 to 400 days (43, 44). The less-generous basic allowance was available for 500 days for those who were not members of an unemployment fund. Thus, due to a gradual loss of benefits, financial hardship may accumulate among the long-term unemployed. Moreover, accumulating unemployment has been linked to lifestyle factors, such as poor diet quality, increased alcohol consumption, and reduced health service use (45, 46), which may adversely affect mental health. Long-term unemployment may also influence health through various other mechanisms such as increased family stress and instability (47).

With all of this in mind, unmeasured confounding may explain some of the association between longer unemployment spells and mental health (2, 5). Previous studies have suggested that stable, individual characteristics such as personality (low emotional self-control, passivity) are particularly important for selection into long-term unemployment (30). We show that controlling for time-invariant characteristics strongly attenuated but did not fully explain the association between current unemployment and mental health. Our results partially support the health-related selection into long-term unemployment hypothesis: Controlling for selection based on time-invariant characteristics fully explained the worse mental health of women with long unemployment histories and notably reduced the impact among men. Few studies have assessed unemployment duration controlling for unobserved confounding, and therefore comparisons are difficult to make. Norwegians who were unemployed for a minimum of 90 days had a slight, gradual decline in their risk of initiating psychotropic medication over the first months of unemployment, followed by a peak at 4 months or more, which indicates a more harmful effect for longer spells of unemployment (*n* = 2,348,552) (36). In partial accordance with our results, these differences were more notable among men than women.

### Variation by sex and age

The reasons behind the gendered impact of unemployment history are unclear. One possible explanation for this relates to income, as men’s salaries often contribute more to family income (2, 48). Even in the context of Finland and Norway, 2 Nordic countries with a relatively high level of female workforce participation (49), men are more likely to be the primary breadwinners (50, 51). Accumulating unemployment is a central threat to material well-being, which may be more damaging to the primary breadwinners. Correspondingly, the mental health gap between the employed and the unemployed is larger in countries with a high proportion of nuclear family households and lower female workforce participation (46, 49). However, other mechanisms are also likely to be at play.

Employment is also a source of social contact, status, and purposeful activities that promote mental health (52–54). The loss of these psychosocial benefits may be more detrimental among men in the long run, as it has previously been suggested that women’s social status, identity, and activities are tied to multiple domains of life, while men’s are more strongly linked to employment (55, 56). Another plausible explanation is that unemployment history may also measure fragmented careers, which may be more the norm among women and thus less damaging to women’s health. Moreover, we showed that current unemployment was associated with poor mental health among both sexes, albeit with relative differences that were stronger among men, which raises the question of why these suggested mechanisms would function similarly for current unemployment but not unemployment histories. More studies are needed to confirm these findings and to address the processes behind them.

Our results also suggest that the association between unemployment and mental health varies by age. In accordance with the literature (5, 31), we did not find unemployment to be less damaging to younger individuals. To the contrary, among men in their 30s, the combination of current unemployment and a longer unemployment history appeared particularly harmful. Meanwhile, among all unemployed women and unemployed men in their 50s, current unemployment but not unemployment history was harmful.

The mechanisms underlying this interplay between age and sex are unclear. The young unemployed may have less savings or be less able to depend on a partner’s income than the older working-age unemployed (57–59). They may less often be eligible for earnings-related unemployment benefits, leading to more rapid financial distress as unemployment cumulates (43, 44). Unemployment may also lead to future wage scarring (60) and hinder accumulating financial assets (e.g., home ownership, savings) (61). Both men and women in their 30s are likely to be starting their careers and families, but men are again more likely to be the main breadwinners (50, 51), and therefore all of the mechanisms together may make younger men more vulnerable to accumulating financial distress while unemployed.

### Methodological considerations

This study is based on large-scale, quarterly, and high-quality register data and a long follow-up. We did not rely on self-reports, which may suffer from higher loss to follow-up, particularly among people with poor mental health (62). Studies based on surveys may also suffer from retrospective biases of past mental health reporting (63, 64), while unemployment histories may be both over and underreported (65).

However, there are limitations related both to the measures and the method in this study. While we analyzed clinically relevant health outcomes, less severe changes in mental health (e.g., less severe depression) as well as untreated mental health problems were not captured by our data. We assume these less severe measures would also reflect changes in employment status, but further studies are needed to evaluate unbiased measures of less severe changes in mental health. For the same reason, even though our employment measures temporally precede the health outcomes, it is possible that we may have not detected prior health deterioration that may have contributed to becoming unemployed. Using a relatively severe measure of mental health, however, may also be an advantage as it is less likely to reflect differences in seeking and access to care.

As we were interested in comparing the unemployed with the employed, we focused on individuals in the labor market. Our exposure measurement was based on registered unemployment, and therefore we were unable to consider discouraged jobless individuals who were no longer registered as unemployed. Moreover, it is unclear whether these individuals should be considered unemployed or outside the labor market, and therefore outside the scope of this study.

Based on these or any other observational analyses of unemployment and mental health, we cannot claim that the association between unemployment and mental health is causal. Although we effectively controlled for all time-invariant confounding (30), time trends, and time-varying partnership status and having children, unmeasured time-varying factors (e.g., changes in social support, behaviors) may still confound the association between unemployment and mental health. In addition, the FE model assumes—somewhat unrealistically—that past health conditions do not affect current unemployment or the time-varying confounders (35). With the FE approach, we also cannot model dynamic relationships between unemployment and health. Lagged health outcomes are endogenous, and we therefore cannot include them in our model. In other words, there is a trade-off between modeling a dynamic association and adjusting for the unobserved, time-invariant characteristics (35); in this paper, we have chosen to focus on the latter.

### Conclusions

We showed that a large part of the difference in mental health between the employed and the unemployed appears to be due to time-invariant confounding. However, the association is not entirely driven by confounding. Even after adjusting for all time-invariant characteristics, current unemployment was associated with a 0.5 percentage-point higher probability of psychiatric conditions and self-harm among both men and women. Among younger men, the deterioration of mental health gained momentum with longer unemployment histories. Selection mechanisms related to stable characteristics are thus likely to explain a large part of the impact of current unemployment on mental health among both men and women, as well as strongly drive the association between long-term unemployment and mental health, particularly among women.

## Supplementary Material

Web_Material_kwac077
