# Supplementary material for: Current Unemployment, Unemployment History, and Mental Health: A Fixed-Effects Model Approach
Source: Am J Epidemiol. 2022 Apr 20;191(8):1459–69. doi: 10.1093/aje/kwac077 (PMC9347014; doi:10.1093/aje/kwac077)
Supplement: Web_Material_kwac077 [file web_material_kwac077.pdf]

WEB MATERIAL

Current Unemployment, Unemployment History and Mental Health: A Fixed Effects Model Approach

Liina Junna, Heta Moustgaard, and Pekka Martikainen.

Table of contents

Web Tables 1–5; pages 2–6

Web Table 1 Sensitivity Analyses: Visits to Specialized Care Due to Psychiatric Conditions and Self-harm Among Finnish Men and Women aged 30-60 in 2008-2018[a]

	2-year unemployment history		4-year unemployment history		5-year unemployment history		Unemployment episodes lasting less than 14 days excluded, 2-year unemployment history	
Exposure	Coef.	95% CI	Coef.	95% CI	Coef.	95% CI	Coef.	95% CI
Men								
Employed	0.0000		0.0000		0.0000		0.0000	
Unemployed	0.0049	0.0047,0.0051	0.0051	0.0049,0.0053	0.052	0.0049,0.0054	0.0053	0.0051,0.0056
Unemployment history, quarters	0.0001	-0.0000,0.0002	-0,0001	-0.0002,-0.0000	-0.0001	-0.0002,-0.0000	-0.0001	-0.0002,0.0000
Unemployment history^2	0.0000	0.0001,0.0000	0.0000	0.0000,0.0000	0.0000	0.0000,0.0000	0.0000	0.0000,0.0000
Women								
Employed	0.0000		0.0000		0.0000			
Unemployed	0.0050	0.0048,0.0053	0.0050	0.0048,0.0053	0.0050	0.0048,0.0053	0.0051	0.0048,0.0053
Unemployment history, quarters	-0,0004	-0.0006,-0.0002	-0,0003	-0.0004,-0.0002	-0,0003	-0.0004,-0.0002	-0.0004	-0.0005,-0.0002
Unemployment history^2	0,0001	0.0000,0.0001	0,0000	0.0000,0.0000	0,0000	0.0000,0.0000	0.0000	0.0000,0.0000

Abbreviations: CI, confidence interval; Coef., coefficient

[a] Results derived from a fixed effects and adjusted for time trend, partnership status, coresident children and all stable characteristics, n = 85,940,600

Web Table 2 Sensitivity Analyses: Logistic Regression, Visits to Specialized Care Due to Psychiatric Conditions and Self-harm Among Finnish Men and Women Aged 30-60 in 2008-2018

	Model 1		Model 2		FE Model 3		FE Model 4	
Exposure	OR	95% CI	OR	95% CI	OR	95% CI	OR	95% CI
Men								
Employed	1.00		1.00		1.00		1.00	
Unemployed	1.51	1.49,1.53	1.57	1.55,1.59	1.49	1.47,1.52	1.49	1.46,1.51
Unemployment history, quarters	1.05	1.05,1.06	0.99	0.99,1.00	0.99	0.99,1.00	0.99	0.99,1.00
Unemployment history^2	1.00	1.00,1.00	1.00	1.00,1.00	1.00	1.00,1.00	1.00	1.00,1.00
N	3,057,092		3,057,092		3,057,092		3,057,092	
Women								
Employed	1.00		1.00		1.00		1.00	
Unemployed	1.38	1.37,1.39	1.46	1.44,1.47	1.39	1.37,1.40	1.38	1.37,1.40
Unemployment history, quarters	1.02	1.01,1.02	0.97	0.97,0.98	0.96	0.96,0.97	0.96	0.96,0.97
Unemployment history^2	1.00	1.00,1.00	1.00	1.00,1.00	1.00	1.00,1.00	1.00	1.00,1.00
N	3,708,973		3,708,973		3,708,973		3,708,973	

Abbreviations: CI, confidence interval; FE, fixed effects; OR, odds ratio.

[a] OLS, current unemployment and unemployment history modelled separately. Adjusted for year, quarter, birth year

[b] OLS, current unemployment and unemployment history modelled together. Adjusted for year, quarter, birth year

[c] FE, adjusted for year, quarter

[d] FE, Model 3 + partnership status and coresident children

Web Table 3 Change in the Probability of Visits to Specialized Care Due to Psychiatric Conditions and Self-harm Among Finnish Men and Women Aged 30–60 From 2008 to 2018[a]

	Men		Women	
Exposure	Coef.	95% CI	Coef.	95% CI
Employed	0.0000		0.0000	
Unemployed	0.0050	0.0047,0.0054	0.0045	0.0040,0.0050
Unemployment history, quarters	0.0000	-0.0001,0.0001	-0.0005	-0.0006,-0.0004
Unemployment history, squared	0.0000	0.0000,0.0000	0.0000	0.0000,0.0000
Employed # unemployment history	0.0000		0.0000	
Unemployed # unemployment history	0.0000	-0.0002,0.0001	0.0004	0.0002,0.0006
Employed # unemployment history, squared	0.0000		0.0000	
Unemployed # unemployment history, squared	0.0000	-0.0000,0.0000	-0.0000	-0.0001,-0.0000
Year	0.0004	0.0004,0.0005	0.0005	0.0004,0.0005
Quarter 1	0.0000		0.0000	
2	0.0002	0.0001,0.0002	0.0001	0.0000,0.0002
3	-0.0002	-0.0003,-0.0002	-0.0009	-0.0009,-0.0008
4	0.0002	0.0001,0.0002	-0.0002	-0.0002,-0.0001
Married, cohabiting	0.0000		0.0000	
Single	0.0001	-0.0002,0.0004	-0.0005	-0.0010,0.0001
Divorced, widowed	0.0024	0.0020,0.0028	0.0037	0.0032,0.0042
No coresident children	0.0000		0.0000	
Coresident children	-0.0017	-0.0019,-0.0015	-0.0005	-0.0008,-0.0003
_cons	-0.8839	-0.9205,-0.8474	-0.9264	-0.9760,-0.8767
rho	0.4649		0.4758	

Abbreviations: CI, confidence interval; Coef., coefficient

[a] Results derived from a fixed effects model of an interaction between current unemployment and unemployment history, n = 85,940,600

Web Table 4 Predicted Probability of Visits to Specialized Care Due to Psychiatric Conditions and Self-harm Among Finnish Men aged 30-60 in 2008-2018[a]

		Ordinary least squares[b]				Fixed effects[c]			
		Employed		Unemployed		Employed		Unemployed	
Age group	Unemployment history, in months	Predicted probability	95% CI	Predicted probability	95% CI	Predicted probability	95% CI	Predicted probability	95% CI
All ages	0	0.006	0.006,0.006	0.018	0.018,0.019	0.008	0.008,0.008	0.013	0.013,0.013
	6	0.009	0.009,0.009	0.022	0.021,0.022	0.008	0.008,0.008	0.013	0.013,0.013
	12	0.011	0.011,0.011	0.024	0.024,0.025	0.008	0.008,0.008	0.013	0.013,0.013
	24	0.013	0.012,0.013	0.028	0.028,0.029	0.009	0.009,0.009	0.014	0.014,0.014
	36	0.013	0.013,0.014	0.031	0.030,0.031	0.010	0.010,0.011	0.016	0.015,0.017
30's	0	0.006	0.006,0.006	0.022	0.021,0.022	0.010	0.010,0.010	0.013	0.013,0.014
	6	0.009	0.009,0.009	0.025	0.025,0.025	0.009	0.009,0.009	0.013	0.012,0.013
	12	0.012	0.011,0.012	0.029	0.029,0.029	0.009	0.009,0.009	0.013	0.012,0.014
	24	0.016	0.015,0.016	0.039	0.038,0.039	0.010	0.009,0.010	0.015	0.014,0.016
	36	0.018	0.017,0.020	0.049	0.047,0.050	0.012	0.010,0.013	0.019	0.017,0.021
40's	0	0.006	0.006,0.006	0.019	0.018,0.020	0.008	0.008,0.008	0.012	0.012,0.013
	6	0.008	0.008,0.008	0.022	0.022,0.023	0.008	0.008,0.008	0.012	0.012,0.013
	12	0.010	0.009,0.010	0.025	0.024,0.026	0.008	0.008,0.008	0.012	0.012,0.013
	24	0.013	0.012,0.013	0.030	0.029,0.031	0.008	0.008,0.009	0.013	0.012,0.014
	36	0.014	0.013,0.015	0.033	0.032,0.035	0.009	0.008,0.011	0.014	0.013,0.016
50's	0	0.005	0.005,0.005	0.015	0.015,0.016	0.007	0.006,0.007	0.011	0.010,0.011
	6	0.006	0.006,0.007	0.016	0.016,0.017	0.007	0.007,0.007	0.011	0.010,0.011
	12	0.007	0.007,0.008	0.017	0.017,0.018	0.007	0.007,0.008	0.011	0.010,0.011
	24	0.009	0.008,0.009	0.019	0.019,0.020	0.008	0.008,0.009	0.011	0.010,0.011
	36	0.010	0.009,0.011	0.020	0.020,0.021	0.008	0.007,0.009	0.011	0.010,0.011

Abbreviations: CI, confidence interval

[a] Results derived from a model of an interaction between current unemployment and unemployment history, n = 43,546,725

[b] Model adjusted for year, quarter, birth year

[c] Model adjusted for year, quarter, square of unemployment history, partnership status and coresident children

Web Table 5 Predicted Probability of Visits to Specialized Care Due to Psychiatric Conditions and Self-harm Among Finnish Women aged 30-60 in 2008-2018[a]

		Ordinary least squares[b]				Fixed effects[c]			
		Employed		Unemployed		Employed		Unemployed	
Age group	Unemployment history, in months	Predicted probability	95% CI	Predicted probability	95% CI	Predicted probability	95% CI	Predicted probability	95% CI
All ages	0	0.010	0.010,0.010	0.026	0.025,0.026	0.012	0.012,0.012	0.017	0.016,0.017
	6	0.013	0.013,0.014	0.028	0.028,0.028	0.012	0.011,0.012	0.017	0.016,0.017
	12	0.016	0.016,0.016	0.030	0.029,0.030	0.011	0.011,0.011	0.017	0.016,0.017
	24	0.017	0.017,0.018	0.031	0.030,0.031	0.011	0.011,0.012	0.017	0.016,0.017
	36	0.014	0.013,0.015	0.029	0.029,0.030	0.013	0.012,0.014	0.017	0.016,0.018
30's	0	0.012	0.012,0.013	0.029	0.028,0.030	0.016	0.016,0.017	0.019	0.018,0.020
	6	0.016	0.016,0.017	0.032	0.031,0.032	0.015	0.015,0.016	0.019	0.018,0.019
	12	0.019	0.019,0.020	0.035	0.034,0.036	0.015	0.014,0.015	0.019	0.018,0.020
	24	0.023	0.023,0.024	0.043	0.042,0.044	0.015	0.014,0.015	0.019	0.018,0.020
	36	0.024	0.022,0.026	0.053	0.051,0.056	0.016	0.013,0.018	0.020	0.017,0.022
40's	0	0.010	0.010,0.010	0.027	0.025,0.028	0.016	0.016,0.017	0.019	0.018,0.020
	6	0.013	0.012,0.013	0.029	0.028,0.029	0.015	0.015,0.016	0.019	0.018,0.019
	12	0.015	0.014,0.015	0.030	0.029,0.031	0.015	0.014,0.015	0.019	0.018,0.020
	24	0.017	0.016,0.018	0.033	0.032,0.034	0.015	0.014,0.015	0.019	0.018,0.020
	36	0.017	0.015,0.019	0.034	0.032,0.035	0.016	0.013,0.018	0.020	0.017,0.022
50's	0	0.008	0.008,0.008	0.021	0.020,0.022	0.009	0.009,0.009	0.014	0.013,0.015
	6	0.010	0.009,0.010	0.021	0.020,0.022	0.009	0.009,0.009	0.013	0.013,0.014
	12	0.010	0.010,0.011	0.021	0.021,0.022	0.009	0.008,0.009	0.013	0.012,0.013
	24	0.011	0.010,0.011	0.021	0.020,0.022	0.009	0.008,0.009	0.012	0.011,0.013
	36	0.008	0.007,0.010	0.020	0.019,0.020	0.010	0.008,0.011	0.012	0.011,0.013

Abbreviations: CI, confidence interval

[a] Results derived from a model of an interaction between current unemployment and unemployment history, n = 42,393,875

[b] Model adjusted for year, quarter, birth year

[c] Model adjusted for year, quarter, square of unemployment history, partnership status and coresident children
